# Supplementary material for: Interface-induced dual-pinning mechanism enhances low-frequency electromagnetic wave loss
Source: Nat Commun. 2024 Apr 17;15:3299. doi: 10.1038/s41467-024-47537-5 (PMC11024160; doi:10.1038/s41467-024-47537-5)
Supplement: Supplementary file 1 — Supplementary Information [file 41467_2024_47537_MOESM1_ESM.pdf]

Supporting Information for Interface-induced Dual-pinning Mechanism Enhances Low-Frequency Electromagnetic Wave Loss

Bo Cai[1], Lu Zhou[1], Pei-Yan Zhao[1], Hua-Long Peng[1], Zhi-Ling Hou[2], Pengfei Hu[3*], Li-Min Liu[4]* and Guang-Sheng Wang[1*]

[1] *School of Chemistry, Beihang University, Beijing 100191, China*

[2] *College of Mathematics and Physics & Beijing Key Laboratory of Environmentally Harmful Chemical Analysis, Beijing University of Chemical Technology, Beijing 100029, China*

[3] *Research Institute of Aero-Engine, Beihang University, Beijing 100191, China*

[4] *School of Physics, Beihang University, Beijing 100191, China*

Email: wanggsh@buaa.edu.cn, hupengfei@buaa.edu.cn, liminliu@buaa.edu.cn

This PDF file includes:

Supplementary Figures 1-19;

Supplementary Table 1, 2;

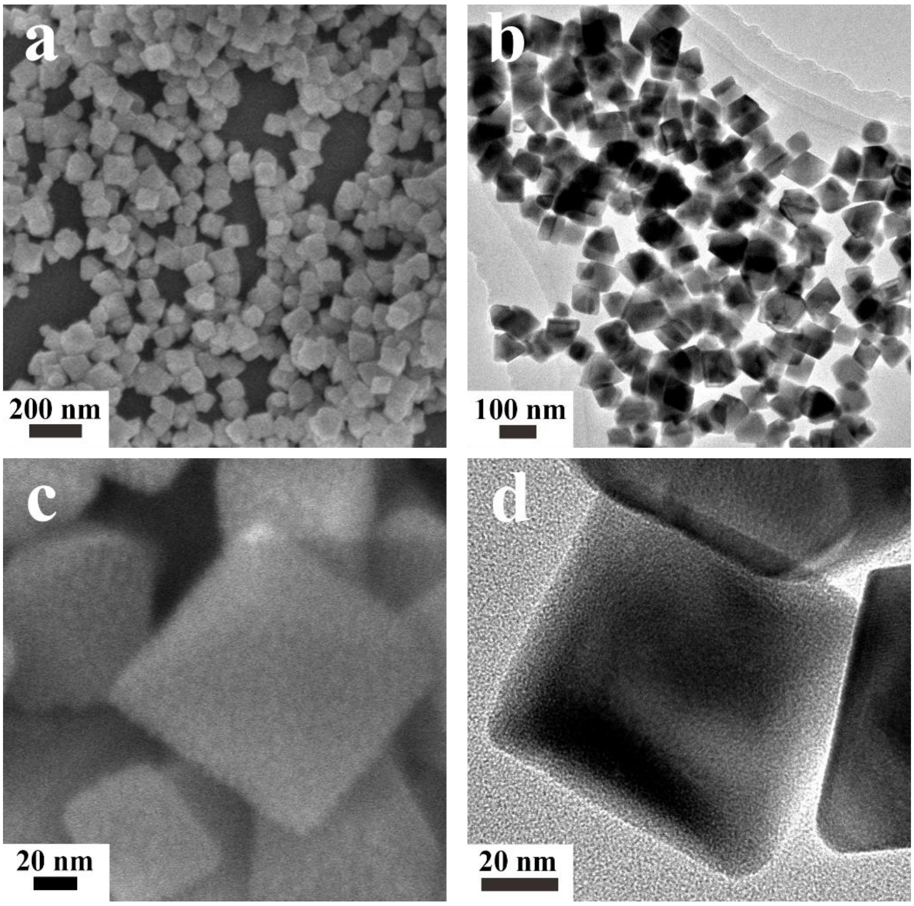

Supplementary Fig. 1 SEM (**a, c**) and TEM (**b, d**) images of $NiFe_2O_4$.

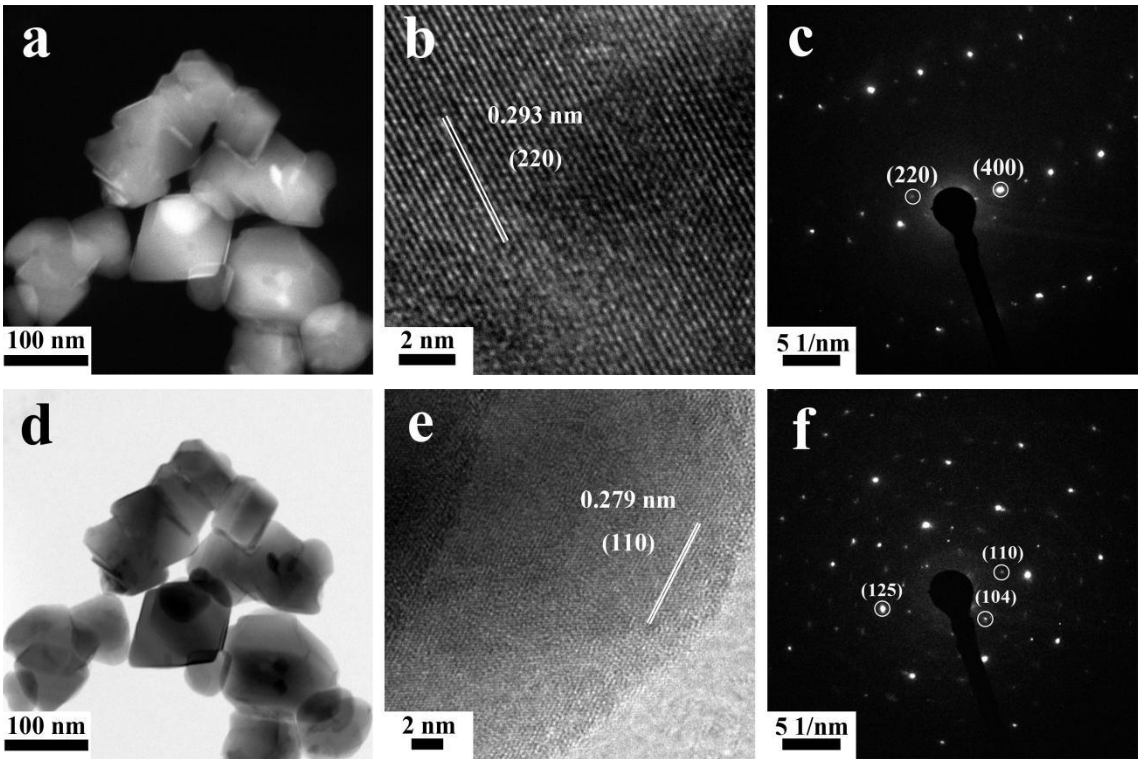

Supplementary Fig. 2 TEM (**a, d**) images of $NiFe_2O_4$@$BiFeO_3$. HRTEM image of a single NFO NP (**b**) and is its corresponding SAED pattern (**c**). HRTEM image of a core-shell NP showing the BFO shell region (**e**) and its corresponding SAED pattern (**f**).

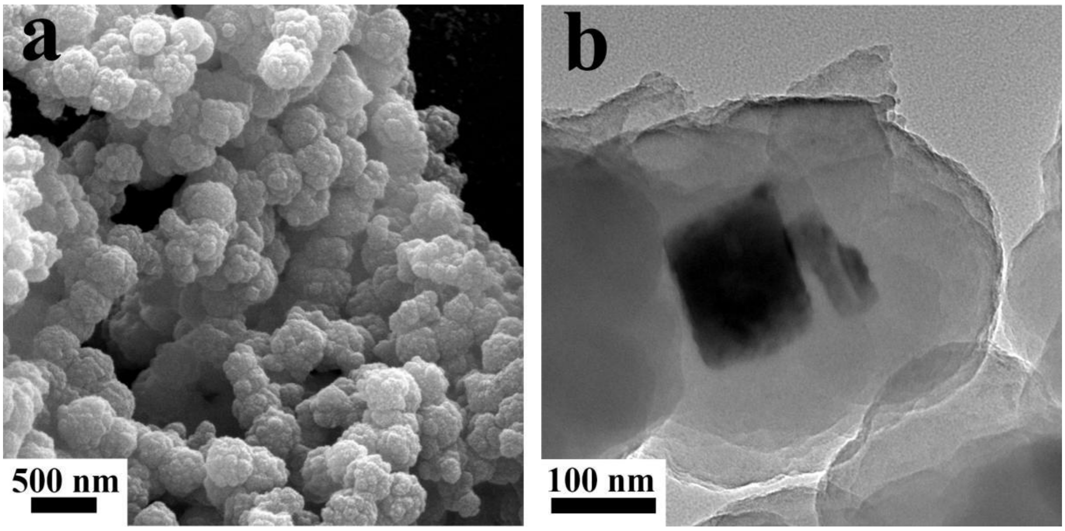

Supplementary Fig. 3 SEM (**a**) and TEM (**b**) images of $NiFe_2O_4$@$BiFeO_3$@PPy.

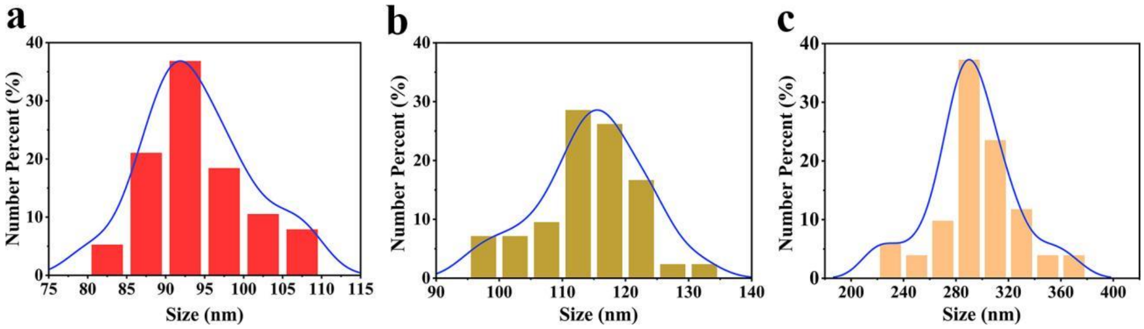

Supplementary Fig. 4 Size distribution of (**a**) NFO NPs with an average size of 94±6 nm, (**b**) NFO@BFO NPs with an average size of 114±8 nm and (**c**) NFO@BFO@PPy NPs with an average size of 0.292±0.032 μm. Source data are provided as a Source Data file.

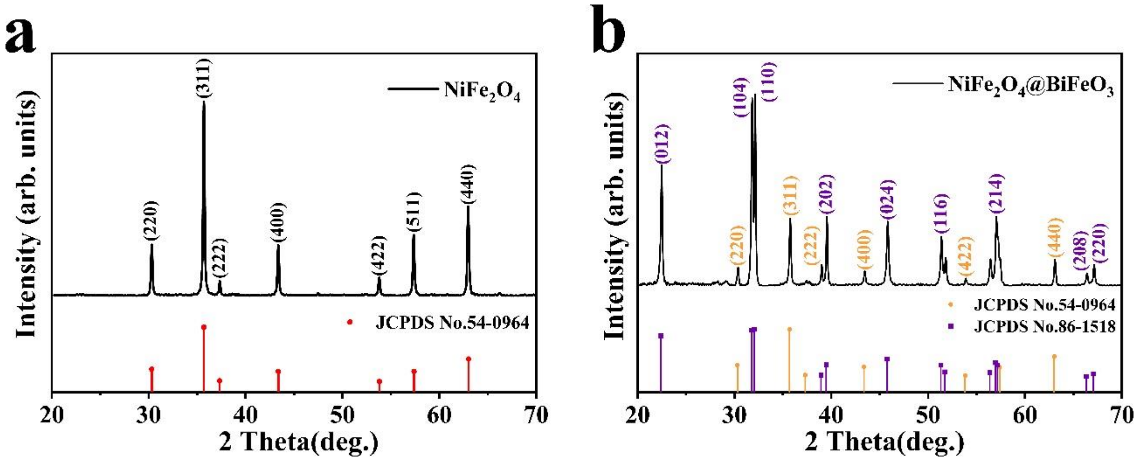

Supplementary Fig. 5 XRD patterns obtained for NFO NPs (**a**) and NFO@BFO NPs (**b**). Source data are provided as a Source Data file.

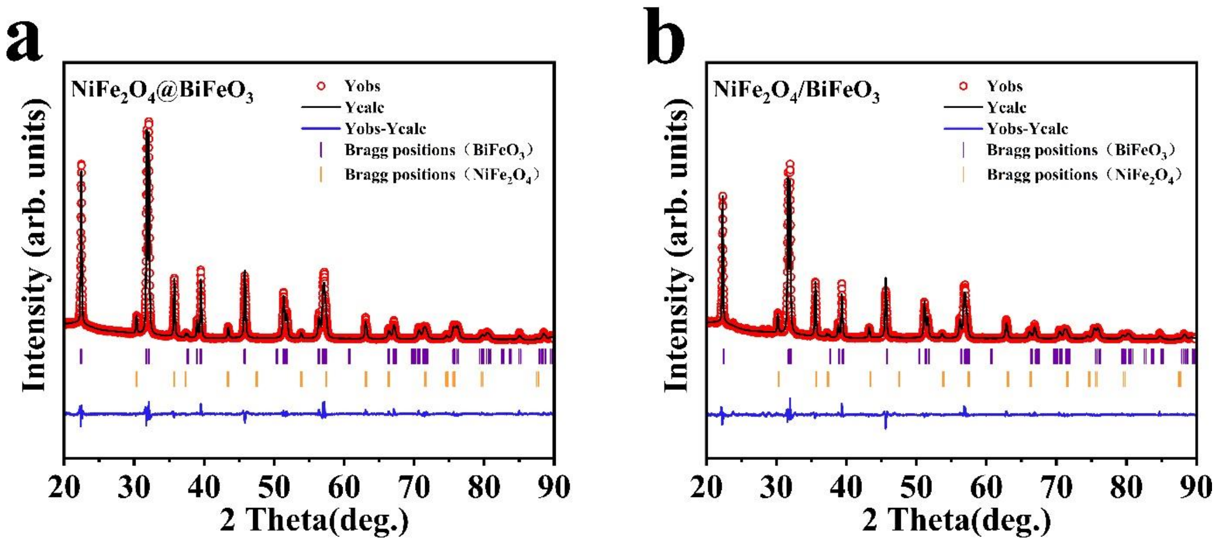

Supplementary Fig. 6 Rietveld refinement results for NFO NPs (**a**) and NFO@BFO NPs (**b**). Source data are provided as a Source Data file.

Supplementary Table. S1 Crystallographic parameters obtained by using Rietveld refinement.

	**A(Å)**		**Calculated density(g/cm³)**		**Fraction(%)**	
	NFO	BFO	NFO	BFO	NFO	BFO
**NFO@BFO**	8.34	5.58(a)/13.87(c)	5.75	8.59	34.7	65.3
**NFO/BFO**	8.34	5.58(a)/13.87(c)	5.50	8.73	40.1	59.9

The SEM and the TEM images exhibit the structures of the samples (Supplementary Figs. 1-3). The NFO NPs prepared in this study were octahedral with an average size of 94 nm. the average size of the monolayer core-shell NFO@BFO NPs was 114 nm. the average size of the final product bilayer core-shell NFO@BFO@PPy NPs was 292 nm (Supplementary Fig. 4). XRD patterns displayed the crystalline structure (Supplementary Fig. 5, Fig 1b). The diffraction peaks of NFO match the (111) (220) (311) (222) (400) (440) (511) crystal planes with Fd3m structure of NFO (JCPDS No. 54-0964), indicating that NFO is a cubic spinel structure. Do the same analysis for NFO@BFO NPs, the independent diffraction peaks correspond to two physical phases, and in addition to the pure NFO diffraction peaks, new peaks of the pure phase of BFO could be assigned to the rhombohedral perovskite structure (JCPDS No. 86-1518), indicating that it has the R3c space group. NFO@BFO@PPy NPs diffraction peaks are essentially the same as those of the NFO@BFO NPs sample, demonstrating that the cladding of polypyrrole does not affect the physical appearance of the core. Crystallographic parameters obtained by using the Rietveld refinement and the fraction of the refinement are shown in Supplementary Fig. 6, Table S1, which is able to verify a similar proportion of components in physically co-mingled NFO/BFO and NFO@BFO composites. Meanwhile, combining TEM and XRD validation results. The High-resolution TEM (HRTEM) analysis and selected area electron diffraction (SAED) pattern of NFO are show in Fig. S2. It is clear that the d-Pitch of 0.293 nm is matched to the (220) crystal plane which belong to the ordered single-crystal structure of NFO core, and the spots in SAED pattern present the Fd3m structure of NFO. Moreover, the BFO is rhombohedral perovskite structure (R3c), the d-spacing of 0.279 nm is matched to the (110) crystallographic plane of the BFO.

Fourier transform infrared (FTIR)

The FTIR spectrum of PPy (Fig. 1c), shows the peak at 1554.8 cm^{-1} corresponds to the $C = C$ stretching vibrations in PPy. FTIR spectra of PPy exhibiting a peak at 1310.3 cm^{-1} which is due to the $C - N$ stretching vibration in PPy. The $N - H$ stretching vibration can be assigned to the peak observed at around 1184.8 cm^{-1}. In plane $C - H$ mode (1044.1 cm^{-1}) and out of plane $C - H$ mode (907.5 cm^{-1}) are due to the PPy polymer chains.

Snoek limit

The real part of the material's permeability inevitably drops abruptly in the GHz bands, while the imaginary part shows a resonance peak, i.e., the Snoek limit：

$$(\mu_i - 1) \cdot f_r = \frac{\gamma}{3\pi} \cdot 4\pi M_s \quad (1)$$

where μ_i is the initial magnetization rate, f_r is the natural resonance frequency, γ is the spin-magnetization ratio, and M_s is the saturation magnetization intensity.

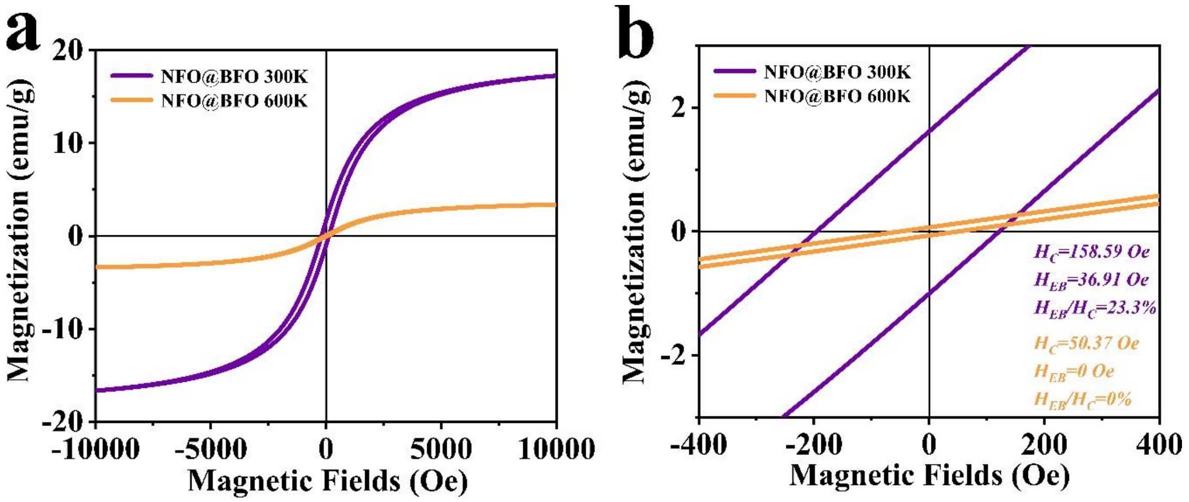

Supplementary Fig. 7 Comparison of *M-H* curves (**a**) and enlarged *M-H* curves (**b**) for NFO@BFO at 300 K and 600 K. NFO@BFO possesses a hysteresis loop displaced along the magnetic field axis at room temperature. Source data are provided as a Source Data file.

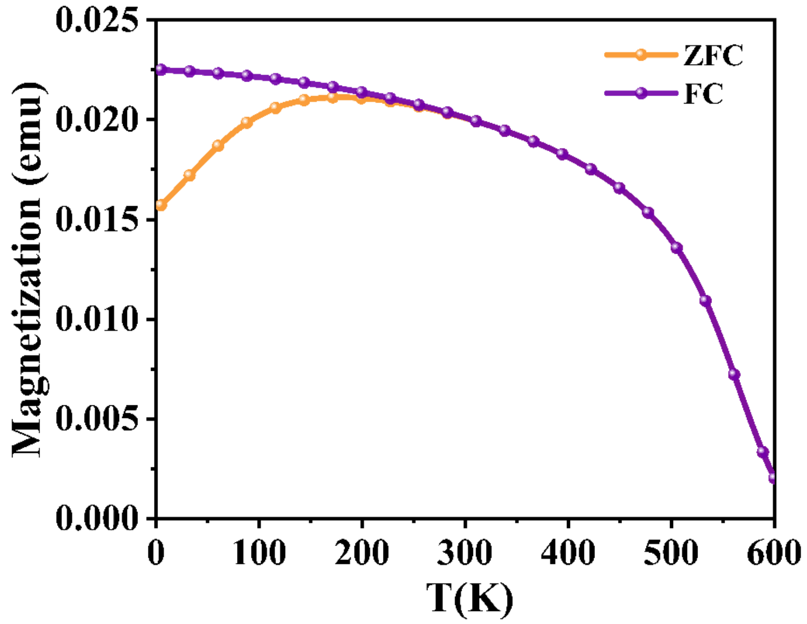

Supplementary Fig. 8 Temperature dependence of ZFC and FC magnetizations observed over the NFO@BFO NPs. Source data are provided as a Source Data file.

The bifurcation of ZFC and FC curves indicates the coexistence of FM and AFM phases in the substance, and their inconsistency reflects the pinning effect of the AFM phase on the FM phase. Above 305 K, the magnetization shows reversible behavior, indicating that all particles are in the superparamagnetic (SP) state. According to the distribution of the blocking temperature (T_B), below this temperature, the ZFC and FC curves split, indicating that the particle system enters the blocking region and the room temperature exchange bias phenomenon exists.

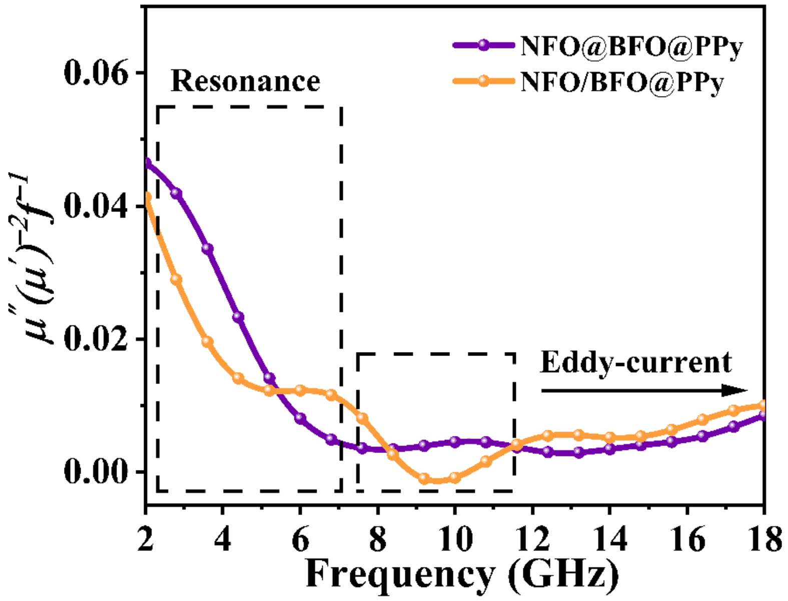

Supplementary Fig. 9 The $\mu''(\mu')^{-2}f^{-1}$ values of NFO@BFO@PPy and NFO/BFO@PPy. Source data are provided as a Source Data file.

Generally, eddy current is also an important factor causing magnetic loss, the mechanism is especially taken effect in these magnetic absorbers with relatively high conductivity. If the $\mu''(\mu')^{-2}f^{-1}$ value of material keeps constant with increasing frequency, then, eddy current loss exists, this mechanism is especially taken effect in these magnetic absorbers with relatively high conductivity. In Supplementary Fig. 8, $\mu''(\mu')^{-2}f^{-1}$ values of all composite samples decrease with the augmentation of the frequency, and the constants do not appear in 2-8 GHz, thus, the magnetic loss from the eddy current can be excluded. It is proved that the magnetic loss of the samples derive from the resonance loss in the low frequency bands.

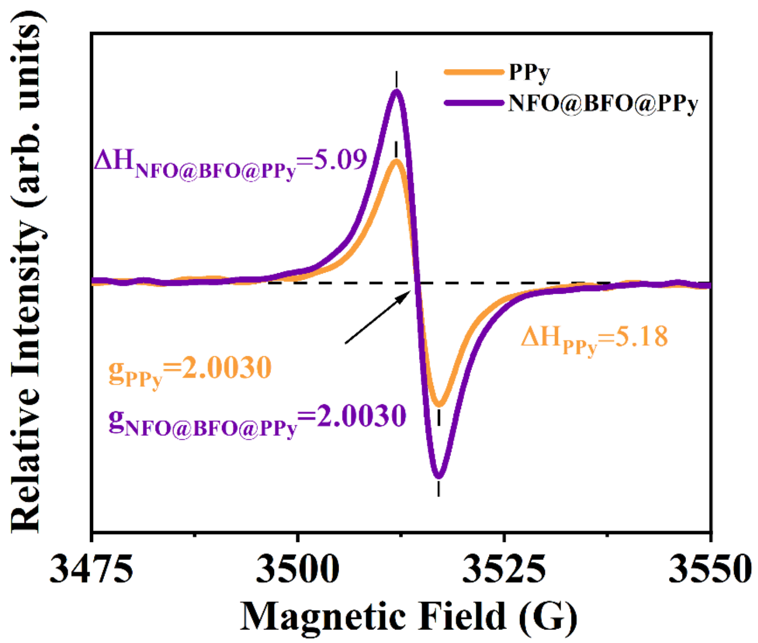

Supplementary Fig. 10 EPR result curve of NFO@BFO@PPy and pure phase polypyrrole. Source data are provided as a Source Data file.

The NFO@BFO@PPy NPs was subjected to EPR analysis with pure phase polypyrrole to verify the dielectric loss mechanism. the EPR parameters obtained from the figure for *Hpp*, *A/B* ratio, and *g*-factor are shown in the table. The EPR spectrum shows a single narrow symmetric EPR signal indicating the presence of free electrons. The stabilized *g*-factor of PPy with NFO@BFO@PPy indicates that the spins on the ring few carbon atoms is off-domain, since the *g*-value of the electrons near the carbon-hydrogen bond is 2.0031. The figure also gives the *A/B* ratio of the EPR spectra of PPy versus NFO@BFO@PPy. The *A/B* asymmetry ratio remains constant for all PPy samples, indicating that the spins are of the free-electron type. The width of the EPR signal is related to the degree of delocalisation of unpaired electrons along the polymer chain. The line width of NFO@BFO@PPy is smaller than that of pure phase PPy, indicating that the PPy shell electrons in the NFO@BFO@PPy sample are more delocalised and more easily polarised.

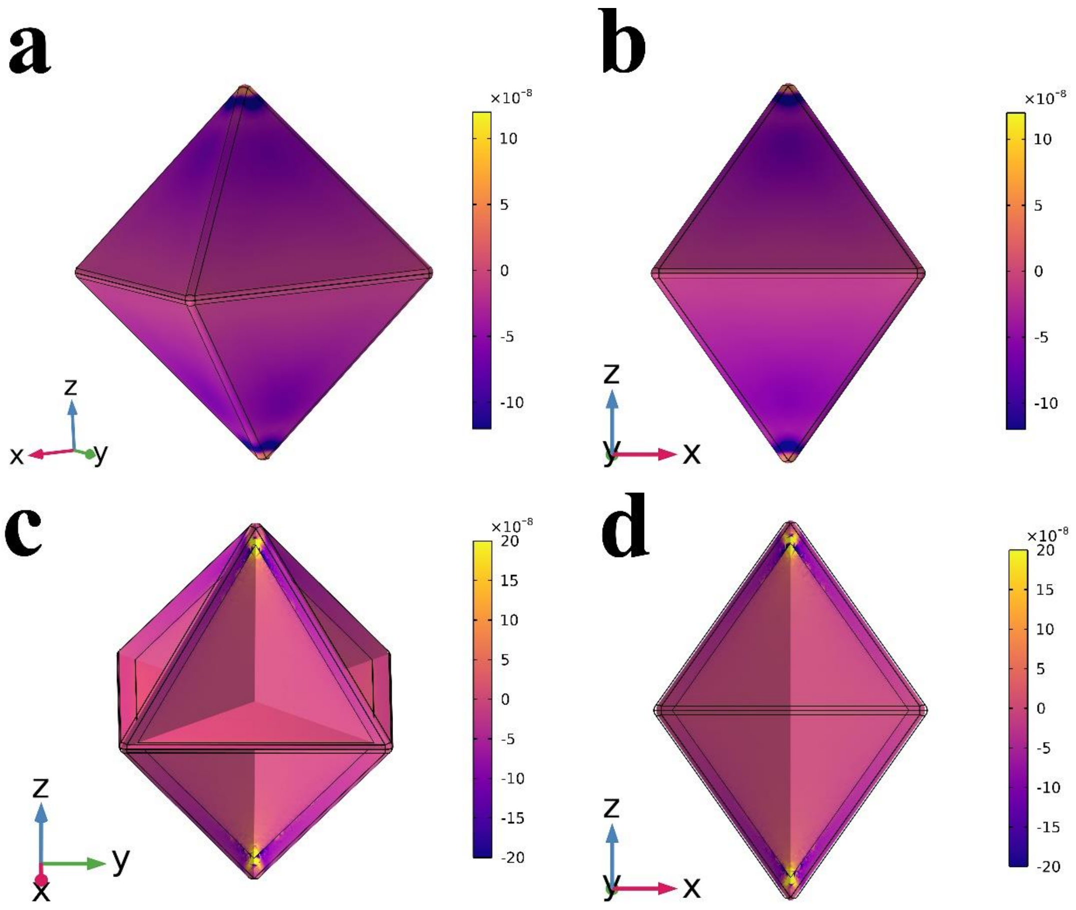

Supplementary Fig. 11 COMSOL Multiphysics simulations on NFO@BFO NPs for a BFO shell thickness of 20 nm and an magnetic field of 3.0 mT. (**a,b**) volumetric strain distribution on the surface of NFO@BFO NP and (**c,d**) the corresponding strain distribution in cross-section.

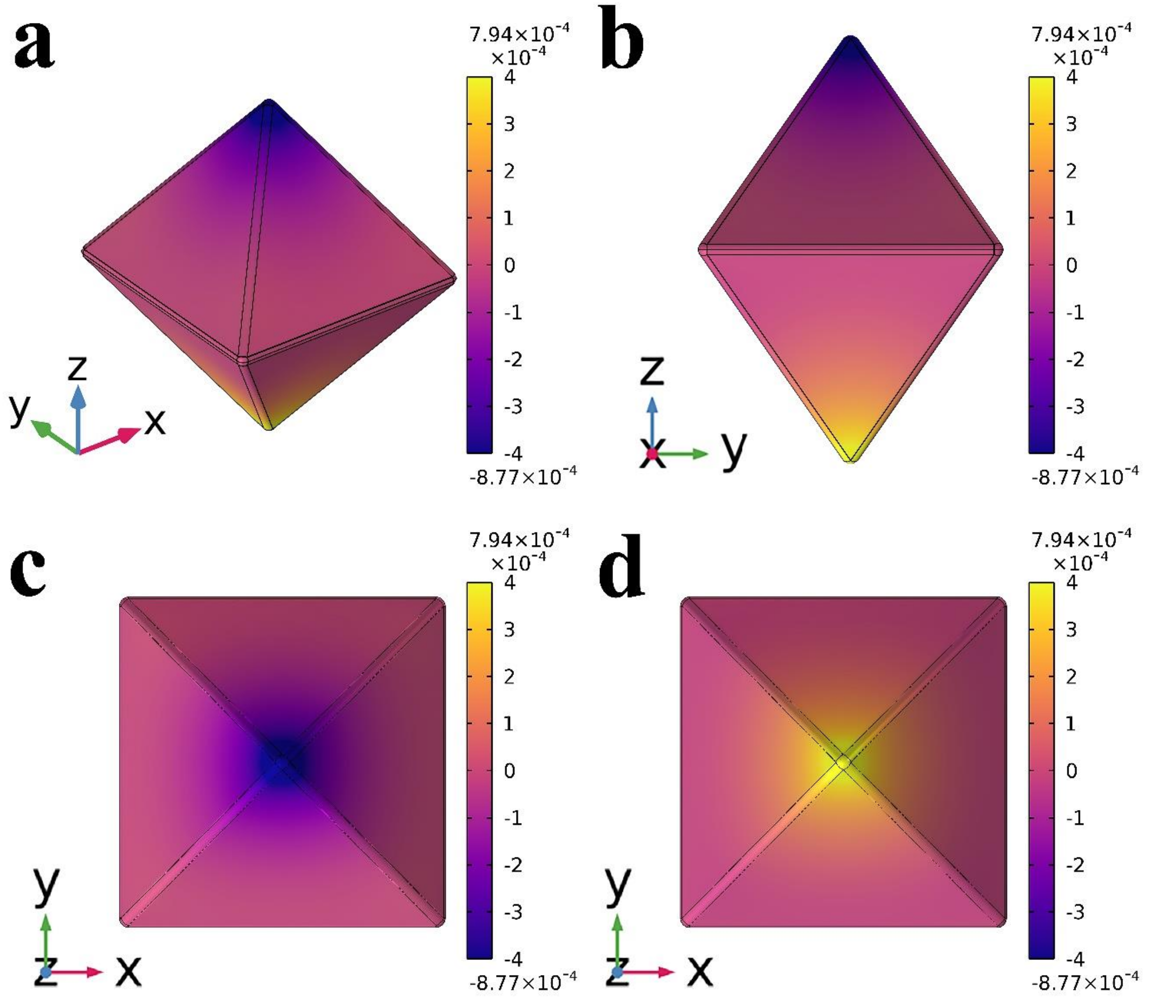

Supplementary Fig. 12 COMSOL Multiphysics simulations on NFO@BFO NPs for a BFO shell thickness of 20 nm and an applied magnetic field of 3.0 mT. Potential generated (**a**) in the cross-section of NFO@BFO NP, (**b-d**) on the surface of BFO shell viewed from different angles showing a gradient potential distribution with opposite polarities on the extreme sides.

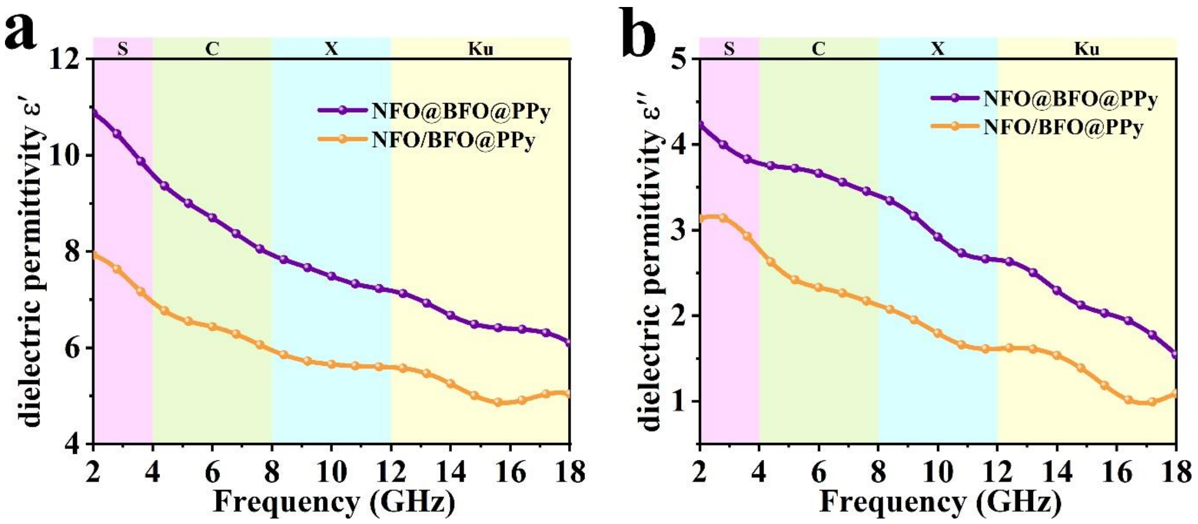

Supplementary Fig. 13 The ε' (**a**) and ε'' (**b**) of NFO@BFO@PPy and NFO/BFO@PPy. Source data are provided as a Source Data file.

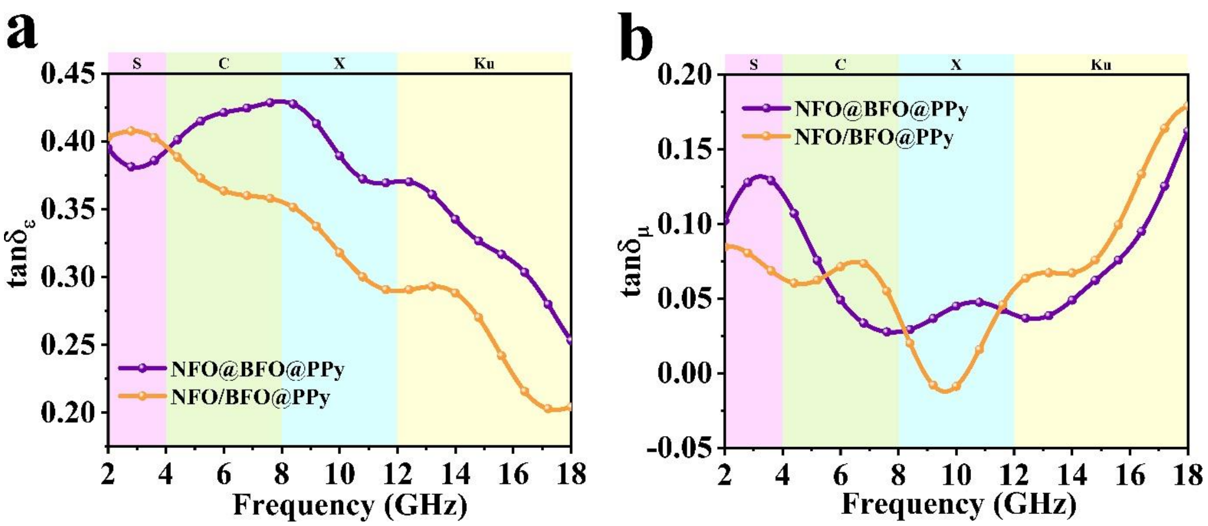

Supplementary Fig. 14 The tan δ_{ε} (**a**) and tan δ_{μ} (**b**) of NFO@BFO@PPy and NFO/BFO@PPy. Source data are provided as a Source Data file.

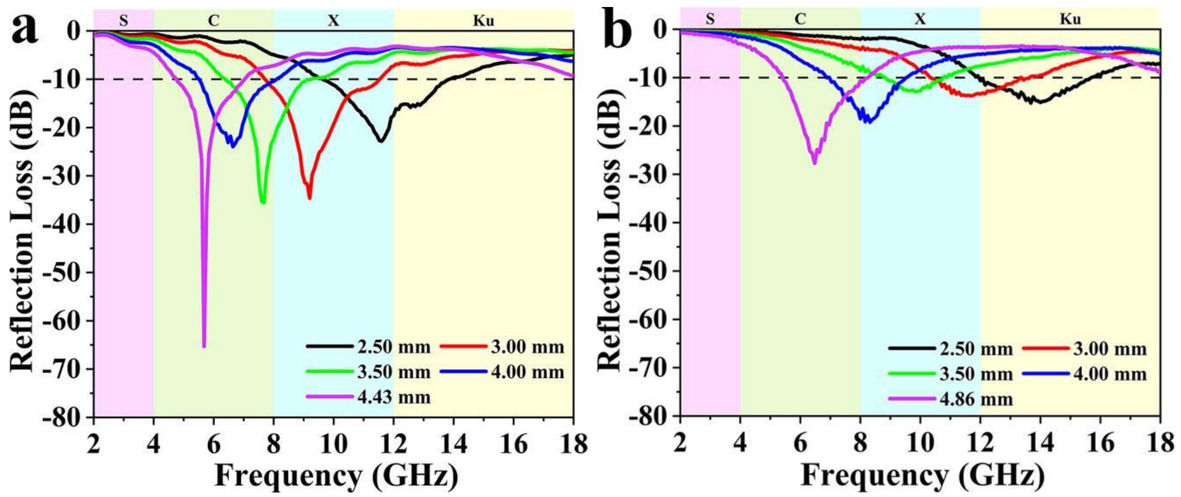

Supplementary Fig. 15 The reflection loss of NFO@BFO@PPy (**a**) and NFO/BFO@PPy (**b**). Source data are provided as a Source Data file.

The RL_{min} of -65.30 dB (the optimal absorption efficiency of 99.99997%) at 5.68 GHz can be achieved for NFO@BFO@PPy composite with the thickness of 4.43 mm. NFO/BFO@PPy composite reaches a RL_{min} of -27.72 dB (the optimal absorption efficiency of 99.831%) at 6.48 GHz and a corresponding thickness of 4.86 mm.

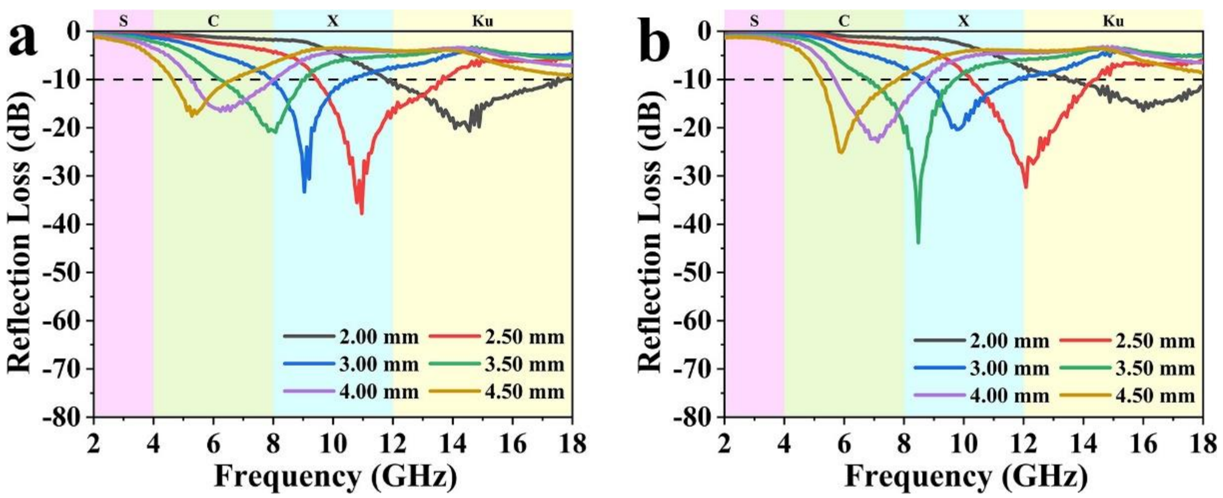

Supplementary Fig. 16 The reflection loss of NFO@PPy (**a**) and BFO@PPy (**b**). Source data are provided as a Source Data file.

The RL_{min} of -37.77 dB (the optimal absorption efficiency of 99.98%) for NFO@PPy composite is achieved at 10.96 GHz with the thickness of 2.50 mm. BFO@PPy composite reaches a RL_{min} of -43.84 dB (the optimal absorption efficiency of 99.996%) at 8.48 GHz and a corresponding thickness of 3.50 mm. From the results of the absorption of electromagnetic waves by a single core component, neither possesses an effective loss of low-frequency electromagnetic waves.

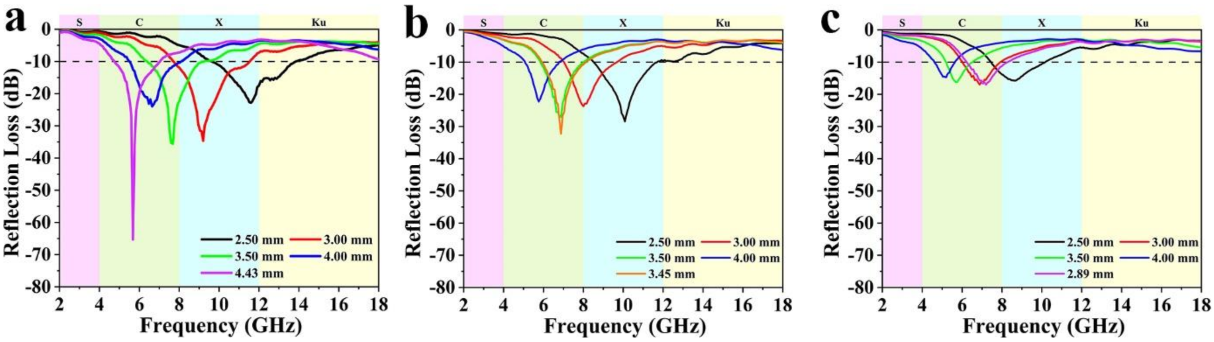

Supplementary Fig. 17 The reflection loss of NFO@BFO@PPy with a fill rate of 15.0% (**a**), 20.0% (**b**), 25.0% (**c**). Source data are provided as a Source Data file.

The NFO@BFO@PPy with a fill rate of 15.0% exhibits the RL_{min} of -65.30 dB at 5.68 GHz, corresponding to a thickness of 4.43 mm. The impedance mismatching is caused by the larger fill rate increasing the dielectric constant of the composite, thus, the RL_{min} of NFO@BFO@PPy with a fill rate of 20.0% at 6.88 GHz is -32.27 dB (the optimal absorption efficiency of 99.94%) and the matching thickness is 3.45 mm, while NFO@BFO@PPy with a fill rate of 25.0% reaches a RL_{min} of -17.01 dB (the optimal absorption efficiency of 98.00%) at 7.20 GHz and a corresponding thickness of 2.89 mm.

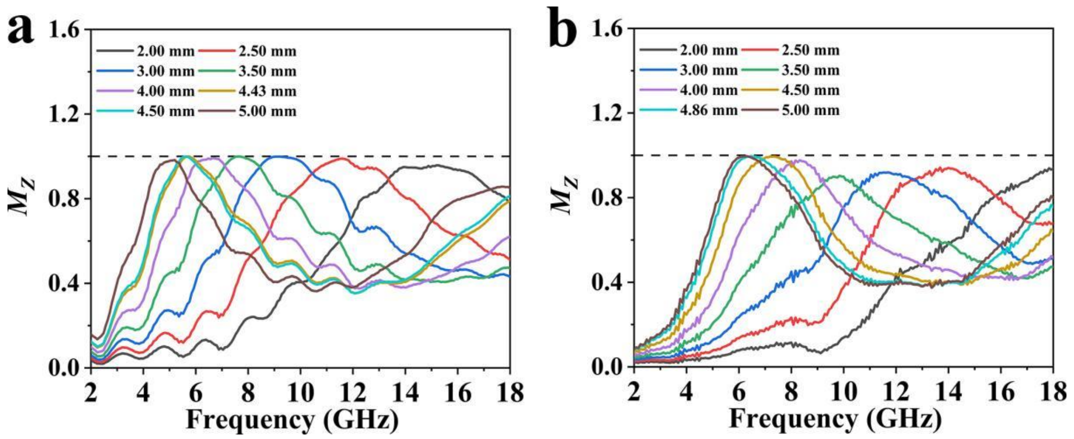

Supplementary Fig. 18 The impedance matching values of NFO@BFO@PPy (**a**) and NFO/BFO@PPy (**b**). Source data are provided as a Source Data file.

The NFO@BFO@PPy composite has the best impedance matching with the M_Z value of 1 at 5.68 GHz. NFO/BFO@PPy composite has the best impedance matching with the M_Z value of 1 at 6.48 GHz.

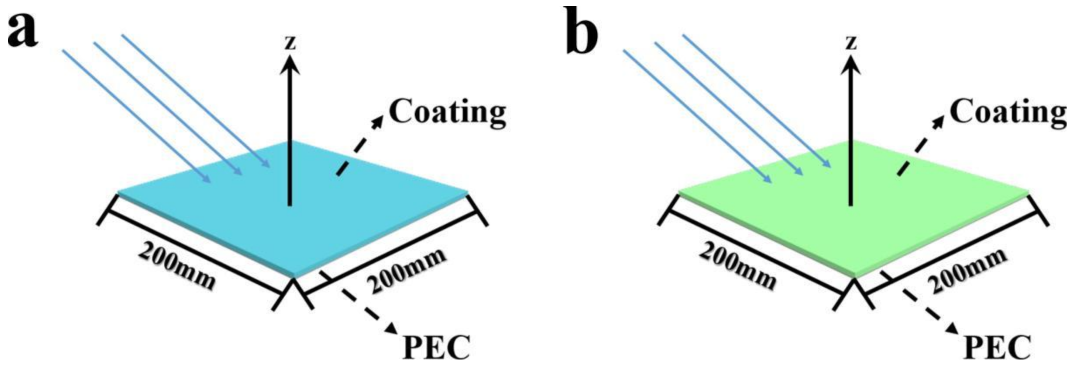

Supplementary Fig. 19 Rectangle PEC covered with coating layer of absorber NFO@BFO@PPy (**a**) and NFO/BFO@PPy (**b**).

Supplementary Table. S2 **EWMA performance in low-frequency bands of typical lower-frequency absorbers.**

Filler	RL_{min} (dB)	EAB (GHz)	f_m (GHz)[a]	Thickness (mm)	Filler loadong (wt%)	Ref.
**$BaFe_{11.6}Co_{0.4}O_{19}$@$Fe_3O_4$**	-48.9	2.5	6.40	3.5	65	**6**
**Graphene–Fe_3O_4**	-40.4	2.2	7.00	5.0	20	**52**
**γ-Fe_2O_3 nanocubes/graphene**	-57.2	1.9	5.12	5.3	20	**53**
**MnO_2@nanoporous carbon**	-57.2	1.4	4.90	4.6	50	**54**
**$NiCo_2O_4$/Co_3O_4/NiO**	-57.0	2.3	4.93	5.9	20	**55**
**Ni@C/G**	-45.5	2.6	6.20	4.8	15	**56**
**Fe_3O_4-Pc@rGO**	-49.0	1.2	5.40	3.8	40	**57**
**$NiFe_2O_4$@$BiFeO_3$@PPy**	-65.3	2.3	5.68	4.4	15	**This Work**

a. f_m: the frequency where RL_{min} is obtained.
